# Controlling malaria in pregnancy: how far from the Abuja targets?

**DOI:** 10.5281/zenodo.10798318

**Published:** 2016-07-08

**Authors:** Oyindamola B. Yusuf, Joshua O. Akinyemi, Adeniyi F. Fagbamigbe, IkeOluwapo O. Ajayi, Elijah A. Bamgboye, Evelyn Ngige, Kawu Issa, Emmanuel Abatta, Onoride Ezire, Perpertual Amida, Adebobola Bashorun

**Affiliations:** 1 Epidemiology and Medical Statistics Unit, Institute of Advanced Medical Research and Training, College of Medicine, University of Ibadan, Nigeria; 2 Department of Epidemiology and Medical Statistics, College of Medicine, University of Ibadan, Nigeria; 3 National AIDS/STD Control Programme, Federal Ministry of Health, Abuja, Nigeria

## Abstract

**Background:**

The Roll Back Malaria (RBM) initiative recommended that all pregnant women receive Inter mittent Preventive Treatment (IPTp) and that by 2010 at least 80% of people at risk of malaria (including pregnant women) use insecticide-treated bednets (ITN) in areas with stable transmission. We evaluated ITN/IPTp coverage, explored its associated factors, and estimated the number of pregnancies protected from malaria.

**Materials and methods:**

This analysis was based on data from the 2012 National HIV/AIDS and Reproductive Health Survey (NARHS Plus). To assess ITN coverage, we used the population of women that was pregnant (n=22,438) at the time of the survey. For IPTp coverage, we used women that had a live birth in the 5 years preceding the survey (n= 118,187) and extracted the population of pregnant women that, during their last pregnancy, received drugs for protection against malaria. We estimated the number of live births using the projected population of females in each state, population of women of child -bearing age and the total fertility rate. The estimated number of pregnancies covered/protected by ITN and IPTp was obtained from a product of the estimated live births and the reported coverage. Multivariate logistic regression was used to determine factors associated with ITN and IPTp use.

**Results:**

We estimated that there were 5,798,897 live births in Nigeria in 2012, of which 3,537,327 and 2,302,162 pregnancies were protected by ITN and IPTp, respectively. Four of 36 states achieved the 80% RBM target for ITN coverage. No state achieved the 100% target for IPTp. Education and socio-economic status were associated with IPTp use.

**Conclusion:**

ITN cover age was higher than in previous estimates even though it is still below the RBM targets. However, IPTp coverage remained low in 2012 and was not likely to increase to match the 2015 target coverage of 100%.

## 1 Introduction

The burden of malaria infection during pregnancy has been reported by several authors [1-5]. The World Health Organization has recommended the use of insecticide-treated bednets (ITNs) and administration of at least two doses of Intermittent Preventive Treatment (IPTp) with sulfadoxine-pyrimethamine (SP) during pregnancy for control of malaria in areas with stable transmission of *P. falciparum* [6]. In addition, the current policy under the National Malaria Control programme is that women should receive at least two doses of SP (Fansidar). The Nigerian Federal Ministry of Health has also adopted this position in its National Antimalarial Treatment Policy. One of the targets of Roll Back Malaria (RBM) and the Millennium Development Goals (MDGs) is to scale up interventions for the prevention and control of malaria in pregnancy. In pursuit of this, the Federal Government of Nigeria, in collaboration with several partners, distributed ~30 million bednets across the country between May 2009 and February 2011. In addition, the Roll Back Malaria (RBM) initiative recommended that all pregnant women receive IPTp, and that at least 80% of people at risk of malaria use ITNs in areas of high-intensity transmission by 2010, including pregnant women [7].

The WHO also recommends at least four antenatal care (ANC) visits, and each visit offers the opportunity to deliver malaria preventive services. RBM had previously recommended a target of 60% IPTp coverage in 2005, which was increased to 80% in 2010 and thereafter to 100% by 2015 [7]. Achieving these targets has been an illusion, particularly in sub-Saharan Africa, despite the reported use of ANC. The challenges are not very evident but might include poor funding. The review of recent evidence suggests that in sub-Saharan Africa, despite the increased prevalence in *P. falciparum* of molecular markers associated with resistance to SP, IPTp-SP remains effective at preventing peripheral parasitaemia, maternal anae-mia, and clinical malaria during pregnancy, and is associated with reduced neonatal mortality. A study in eastern Nigeria showed that the use of ITNs and SP combined was a more effective method in protecting pregnant women than either ITN or IPTp alone, although the cost of getting the combined treatment might affect its use [8].

Overall, most studies suggest that IPTp with SP remains effective, or at least is not associated with any harm, in areas with high prevalence of quintuple-mutant *P. falci-parum* parasites [6]. Studies have reported ITN use much lower than the RBM targets [9,10]. In Nigeria, ANC use is suboptimal, as the 2012 NARHS survey among pregnant women reported a utilisation rate of 52.5% [11]. In Lagos, Nigeria, ITN coverage was 11.2% and 37.5% in public and in private clinics, respectively [10]. Another study in eastern Nigeria reported an ANC utilisation rate of 20.7% [9]. A review of ITN use during pregnancy showed that it varied from as low as 3% to >80% in African countries [12]. One of the indicators recommended for the assessment of progress in malaria prevention with ITNs has been the proportion of pregnant women sleeping under an ITN the previous night [7]. This indicator has been used to monitor progress of intervention programmes. These intervention programmes usually take place in the health facility where the pregnant woman receives ANC services. As access to ANC is expected to vary by state or community, it is also expected that ITN coverage will vary. Assessing coverage is essential for designing appropriate health programmes and policies in order to meet the MDGs. The reasons why the RBM targets have remained unmet remain unclear. In Nigeria, several studies have assessed ITN coverage and IPTp, but none has used a nationally representative sample. Thus, the generalisability of the results of these studies is limited in a diverse country like Nigeria. It therefore becomes important to analyse ITN/IPTp coverage using nationally representative data. In this study, we report IT-N/IPTp coverage for each state in Nigeria using data from the 2012 NARHS Plus, with the expectation that findings will be used by malaria programme managers and public health practitioners to plan appropriate interventions and delivery to improve coverage of IPTp and ITN use among pregnant women. We explored association between coverage of each intervention and factors that could affect access, such as location of residence, education, ANC utili-sation, geographical location and socioeconomic status (measured by wealth quintile). We also estimated the number of pregnancies covered/protected from malaria, which will be useful for policy makers to determine the need for disease prevention tools for malaria control in pregnancy.

## 2 Materials and methods

This paper is based on data from the 2012 National HIV/ AIDS and Reproductive and Health Survey (NARHS Plus). NARHS 2012 Plus was a cross-sectional study of men and women of reproductive age. A stratified, multistage cluster sampling technique was used to select a nationally representative probability sample of women aged 15-49 years and men aged 15-64 years living in households in rural and urban areas in all 36 states and the Federal Capital Territory (FCT) of Nigeria. Stage 1 involved the selection of rural and urban localities from each state and the FCT. Stage 2 involved the selection of Enumeration Areas (EA) within the selected rural and urban localities. Stage 3 involved the listing and selection of households. Thirty-two households were sampled from each of the 30 sampled EA (clusters) from each state. Overall, 35,520 households were selected for final interview. Details of the methodology have been published previously [11].

We extracted data on the use of ITNs, IPTp and ANC visits. To assess ITN use, we used the population of women that was pregnant (*n*=22,438) at the time of survey, and they were asked whether they slept under an ITN the night preceding the survey. For the assessment of coverage of intermittent preventive treatment, we used women with a live birth in the 5 years preceding the survey (*n*=118,187). We did not include women who were pregnant at the time of survey because they could have received IPTp between the time of survey and their delivery. To assess IPTp use, we used the population of pregnant women that, during their last pregnancy, received drugs for protection against malaria. For ANC utilisation, we used the proportion of pregnant women that received ANC in their previous pregnancy. We determined the percentage of pregnant women protected by IPTp and ITN, and subsequently estimated the number of live births and the number of pregnancies covered. Our main outcome measures for coverage were uptake of IPTp and ITN use. Our explanatory variables included location (rural/urban), geopolitical zone, education (formal/non-formal) and wealth index. The wealth quintiles were based on household assets and were obtained by principal component analysis [13]. Multivariate logistic regression was used to determine factors associated with ITN and IPTp use at 5% level of significance. Model fit was assessed using Hosmer-Lemeshow goodness of fit test. Odds ratios and 95% CI were presented. Stata version 12.0 was used for analysis [14]. The data were weighted to reflect differences in population sizes of the states.

We estimated the number of live births in 2012 for each state as follows:

The proportion of female population in the 2006 census for each state was computed [15]. Using the 2012 projected Nigeria female population from the United Nations world population prospects, the proportion of females from each state was used to estimate the projected female population in each state for 2012.

For each state, the proportion of women of childbearing age (WOCBA) and total fertility rate (TFR) were adapted from the household sample of the 2013 Nigeria Demographic and Health Survey (NDHS) [16]. The number of WOCBA was obtained by multiplying the proportion of WOCBA with the projected population of women.

As the TFR divided by 35 gives the average number of live birth per WOCBA per year, the number of live births in 2012 was calculated for each state as:

No. of livebirth=TFR35x No. of WOCBA

[17].

Finally, the estimated number of pregnancies covered by ITN and IPTp was obtained from a product of the estimated live births and the reported coverage shown in Table 2.

## 3 Results

### 3.1 General characteristics of the respondents

A total of 290,273 records of women aged 15-49 years was used for analysis; 118,187 gave birth in the last 5 years, while 22,438 were pregnant at the time of the survey. The mean age was 29.4 years, SD=9.4. Of the 118,187 women that gave birth in the last 5 years, 62.3% had received formal education, and 66.8% lived in rural areas. Of the 22,438 women that were pregnant at the time of the survey, 64.3% had received formal education, and 67.8% lived in rural areas. Table 1 shows the background characteristics of the two categories of women.

**Table 1.** Socio-demographic characteristics of respondents.

Characteristic	Gave birth in last 5 yrs (n=118,187)	Currently pregnant (n=22,438)
Age: mean (±SD)	28.5 (6.9) yrs	26.7 (6.5) yrs
*Education*		
No formal education	44,565 (37.7%)	805 (35.7%)
Formal education	73,622 (62.3%)	14,433 (64.3%)
*Wealth quintile*		
Poorest	35,361 (29.9%)	6970 (31.1%)
Poorer	25,686 (21.7%)	4695 (20.9%)
Average	19,370 (16.4%)	3854 (17.2%)
Wealthier	18,836 (15.9%)	3338 (14.9%)
Wealthiest	18,900 (16.0%)	3579 (16.0%)
*Location*		
Urban	39,259 (33.2%)	7226 (32.2%)
Rural	78,928 (66.8%)	15,212 (67.8%)
*Geopolitical zone*		
North-central	28,025 (23.7%)	5591 (24.9%)
North-east	18,275 (15.5%)	3112 (13.9%)
North-west	34,656 (29.3%)	7033 (31.3%)
South-east	4876 (4.1%)	960 (4.3%)
South-south	9750 (8.2%)	1694 (7.5%)
South-west	22,605 (19.1%)	4048 (18.0%)

### 3.2 ITN, ANC and IPTp use

**Figure 1.**
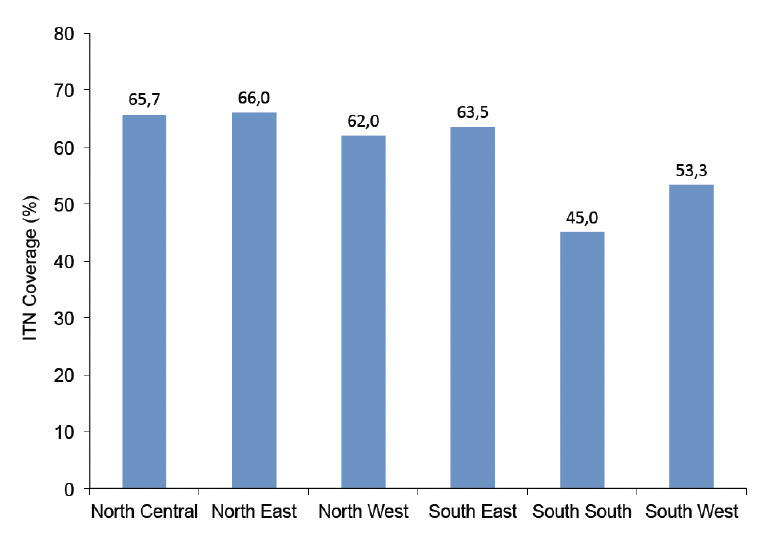
ITN use by geopolitical zone.

**Figure 2.**
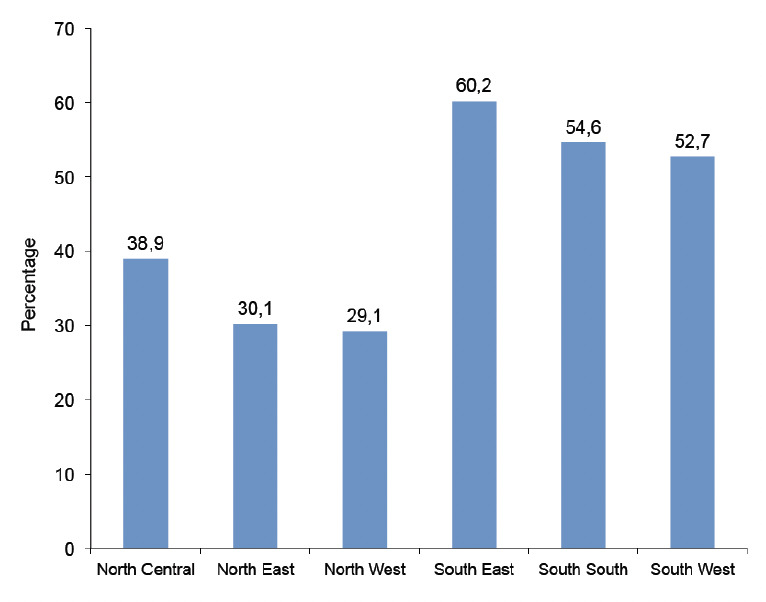
IPTp use by geopolitical zone.

The proportion of women with live births in the last 5 years ranged from 33.7% in Imo state to 73.4% in Yobe state. The number of women receiving ANC ranged from 14.9% in Zamfara to 94.2% in the Federal Capital Territory, Abuja. The percentage of women receiving IPTp varied from as low as 6.5% in Katsina to 71.0% in Abia. The proportion of pregnant women that slept under an ITN the night before the survey ranged from 12.0% in Katsina to >80% in Yobe (Table 2). ITN use was highest in the north -central zone (65.7%) and lowest in the south-south (45.0%), whereas IPTp use was highest in the south-east (60.2%) and lowest in the north-west (29.1%) (Figure 1 and 2).

**Table 2.** Frequency distribution of women (15-49 years) with live births, ANC attendance in last pregnancy, and use of IPTp and ITN, by state.

State	% of women with live birth in last 5 yrs	% of women with ANC in last pregnancy	% of women with IPTp in last pregnancy	% ITN use among pregnant women
ABIA	100 (44.4)	95 (89.6)	66 (71.0)	10 (62.5)
ADAMAWA	254 (44.6)	172 (61.9)	92 (39.3)	58 (70.7)
AKWA IBOM	483 (53.7)	255 (57.0)	219 (50.3)	15 (45.5)
ANAMBRA	608 (52.4)	564 (92.8)	292 (56.2)	52 (68.4)
BAUCHI	1035 (74.2)	585 (63.6)	420 (49.4)	110 (73.3)
BAYELSA	1470 (65.7)	762 (50.8)	720 (55.3)	126 (61.8)
BENUE	1519 (67.8)	896 (62.1)	413 (31.4)	140 (71.4)
BORNO	920 (52.5)	224 (23.5)	120 (12.9)	144 (10.0)
CROSS RIVER	891 (41.4)	729 (77.9)	396 (53.0)	45 (62.5)
DELTA	1950 (63.1)	1430 (76.1)	900 (53.6)	40 (44.4)
EBONYI	1320 (48.4)	990 (72.6)	660 (55.6)	154 (87.5)
EDO	1500 (52.5)	1224 (81.0)	720 (53.6)	156 (50.0)
EKITI	1859 (52.2)	1456 (80.6)	520 (31.3)	104 (66.7)
ENUGU	1792 (50.2)	1638 (86.0)	1092 (61.4)	98 (63.6)
GOMBE	3360 (68.5)	2325 (71.1)	1425 (46.1)	285 (63.3)
IMO	1056 (33.7)	912 (77.0)	688 (64.2)	208 (52.0)
JIGAWA	4760 (71.6)	1955 (40.8)	1122 (26.1)	493 (76.3)
KADUNA	3582 (66.8)	2808 (81.7)	1692 (49.7)	288 (69.6)
KANO	4161 (72.3)	2698 (68.6)	2356 (61.1)	209 (42.3)
KATSINA	4360 (65.5)	1760 (36.5)	280 (6.5)	60 (12.0)
KEBBI	2772 (44.4)	630 (20.7)	336 (12.3)	336 (41.0)
KOGI	3300 (52.8)	2816 (83.1)	1122 (34.7)	66 (50.0)
KWARA	3059 (44.5)	2300 (56.5)	1104 (32.7)	184 (80.0)
LAGOS	4440 (63.1)	3840 (89.4)	2688 (70.4)	312 (54.2)
NASARAWA	3000 (42.9)	1400 (40.9)	1000 (32.0)	575 (79.3)
NIGER	6188 (68.8)	3406 (56.5)	1378 (25.5)	416 (57.1)
OGUN	4077 (49.2)	3510 (87.8)	1998 (63.8)	189 (38.9)
ONDO	3192 (50.9)	2716 (71.9)	1736 (48.1)	140 (35.7)
OSUN	4147 (49.1)	3828 (91.7)	1769 (44.5)	58 (40.0)
OYO	4890 (49.4)	4500 (76.9)	2610 (49.4)	420 (77.8)
PLATEAU	5890 (61.7)	4371 (73.8)	2139 (41.8)	496 (72.7)
RIVERS	3456 (54.5)	2464 (71.3)	1696 (56.4)	96 (27.3)
SOKOTO	6633 (64.4)	1848 (27.2)	2376 (39.1)	495 (75.0)
TARABA	7106 (63.5)	4080 (64.5)	2142 (38.0)	646 (51.4)
YOBE	5600 (73.4)	805 (16.0)	525 (10.6)	525 (88.2)
ZAMFARA	8352 (63.9)	1332 (14.9)	936 (14.2)	1224 (82.9)
FCT	5069 (64.9)	4810 (94.2)	3034 (66.1)	185 (41.7)
Total	118,151 (50.5)	72,134 (59.7)	42,782 (39.7)	9158 (61.0)

**Figure 3.**
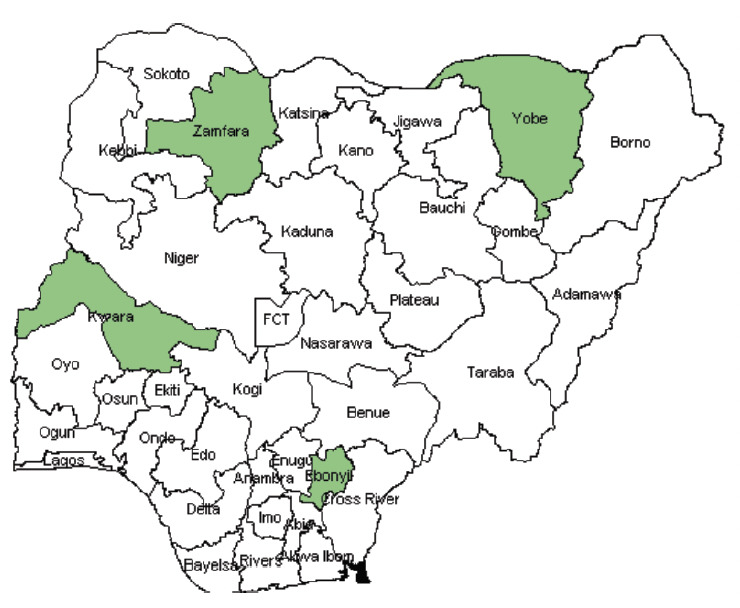
ITN coverage in Nigeria (states shown in green have met the RBM target of 80% coverage).

We estimated a total of 5,798,897 live births in Nigeria in 2012, of which 3,537,327 pregnancies were covered/protected by ITNs and 2,302,162 pregnancies were protected with IPTp. The number of pregnancies covered by ITNs ranged from 14,919 in Borno to 241,570 in Lagos State. The number of pregnancies covered by IPTp ranged from 19,245 in Borno to 130,941 in Zamfara. There were similarities in coverage of ITN and IPTp in states such as Akwa Ibom, Bayelsa, and Enugu. On the other hand, wide gaps were recorded in several states, including Benue, Ekiti, Jigawa, Lagos, Yobe, Zamfara, Taraba, Rivers, Oyo, Niger, Kogi, Katsina, Nasarawa and Plateau (Table 3). Figure 3 shows the map of the country illustrating where the 80% coverage target was met, namely Kwara, Zamfara, Yobe and Ebonyi.

**Table 3.** Estimated/projected number of pregnancies cover ed by ITN and IPTp by state in Nigeria.

State	Female population (2006 Census)	2012 Projected female population	% of WOCBA^1^ (from NDHS^2^ 2013)	TFR^3^ (from NDHS 2013)	WOCBA (2012)	Estimated live births	ITN coverage	IPTp coverage	Estimated number of pregnancies covered	
									ITN	IPTp
ABIA	1,415,082	1,698,558	43.9	4.2	745,667	89,480	62.5	71.0	55,925	63,531
ADAMAWA	1,571,680	1,886,526	43.9	5.8	828,185	137,242	70.7	39.3	97,030	53,936
AKWA IBOM	1,918,849	2,303,242	46.1	3.9	1,061,795	118,314	45.5	50.3	53,833	59,512
ANAMBRA	2,059,844	2,472,482	46.7	4.2	1,154,649	138,558	68.4	56.2	94,774	77,870
BAUCHI	2,283,800	2,741,302	41.7	8.1	1,143,123	264,551	73.3	49.4	193,916	130,688
BAYELSA	830,432	996,788	50.3	4.5	501,384	64,464	61.8	55.3	39,839	35,648
BENUE	2,109,598	2,532,203	44.0	5.2	1,114,169	165,534	71.4	31.4	118,191	51,978
BORNO	2,007,746	2,409,947	46.1	4.7	1,110,986	149,190	10.0	12.9	14,919	19,245
CROSS RIVER	1,421,021	1,705,687	42.8	5.4	730,034	112,634	62.5	53.0	70,396	59,696
DELTA	2,043,136	2,452,427	50.8	4.1	1,245,833	145,940	44.4	53.6	64,798	78,224
EBONYI	1,112,791	1,335,711	45.0	5.3	601,070	91,019	87.5	55.6	79,642	50,607
EDO	1,599,420	1,919,823	48.6	4.4	933,034	117,296	50.0	53.6	58,648	62,871
EKITI	1,183,470	1,420,548	46.0	4.3	653,452	80,281	66.7	31.3	53,548	25,128
ENUGU	1,671,795	2,006,697	45.0	4.8	903,014	123,842	63.6	61.4	78,763	76,039
FCT-ABUJA	673,067	807,899	51.7	4.5	417,684	53,702	63.3	46.1	33,993	24,757
GOMBE	1,120,812	1,345,338	40.1	7.0	539,481	107,896	52.0	64.2	56,106	69,269
IMO	1,951,092	2,341,944	38.9	4.8	911,016	124,939	76.3	26.1	95,329	32,609
JIGAWA	2,162,926	2,596,214	41.1	7.6	1,067,044	231,701	69.6	49.7	161,264	115,155
KADUNA	3,023,065	3,628,660	47.6	4.1	1,727,242	202,334	42.3	61.1	85,587	123,626
KANO	4,453,336	5,345,450	41.1	6.8	2,196,980	426,842	12.0	6.5	51,221	27,745
KATSINA	2,853,305	3,424,893	40.6	7.4	1,390,506	293,993	41.0	12.3	120,537	36,161
KEBBI	1,624,912	1,950,422	40.4	6.7	787,971	150,840	50.0	34.7	75,420	52,342
KOGI	1,641,140	1,969,901	44.2	7.0	870,696	174,139	80.0	32.7	139,311	56,944
KWARA	1,171,570	1,406,264	44.8	5.1	630,006	91,801	54.2	70.4	49,756	64,628
LAGOS	4,394,480	5,274,803	49.3	4.1	2,600,478	304,627	79.3	32.0	241,570	97,481
NASARAWA	925,576	1,110,992	43.4	5.4	482,170	74,392	57.1	25.5	42,478	18,970
NIGER	1,950,422	2,341,140	40.9	6.1	957,526	166,883	38.9	63.8	64,918	106,471
OGUN	1,886,233	2,264,092	41.5	5.4	939,598	144,967	35.7	48.1	51,753	69,729
ONDO	1,715,820	2,059,541	44.4	5.2	914,436	135,859	40.0	44.5	54,344	60,457
OSUN	1,682,810	2,019,919	47.5	4.1	959,461	112,394	77.8	49.4	87,443	55,523
OYO	2,778,462	3,335,057	44.9	4.5	1,497,440	192,528	72.7	41.8	139,968	80,477
PLATEAU	1,607,533	1,929,562	42.6	5.4	821,993	126,822	27.3	56.4	34,622	71,528
RIVERS	2,525,690	3,031,648	53.6	3.8	1,624,963	176,425	75.0	39.1	132,318	68,982
SOKOTO	1,838,963	2,207,353	44.8	7.0	988,894	197,779	51.4	38.0	101,658	75,156
TARABA	1,122,869	1,347,807	47.0	6.0	633,470	108,595	88.2	10.6	95,781	11,511
YOBE	1,116,305	1,339,929	43.5	6.6	582,869	109,912	82.9	14.2	91,117	15,608
ZAMFARA	1,637,250	1,965,232	42.0	8.4	825,397	198,095	41.7	66.1	82,606	130,941
Total	69,086,302	82,926,000	44.5	5.5	36,902,070	5,798,897	61.0	39.7	3,537,327	2,302,162

1. WOCBA: Women of Child Bearing Age; 2. NDHS: Nigeria Demographic and Health Survey; 3. TFR: Total Fertility Rate.

### 3.3 Factors associated with ITN and IPTp use

Based on a multivariate logistic regression model, respondents from the south-south region were less likely to use ITNs compared with those in the north-central (OR=0.459, 95% CI: 0.252-0.837) (Table 4). Respondents from north-east, south-east and south-south were more likely to use IPTp compared with those from the north-central. Formal education was also a predictor of higher IPTp use (OR=1.97, 95%CI: 1.706-2.275). (Table 5).

**Table 4.** Factors associated with ITN use.

	OR	95% CI	P-value
*Education*				
*No formal education				
Formal education	0.845	0.561 - 1.273	0.421
*Wealth quintile*				
*Poorest				
Poorer	1.138	0.714 - 1.814	0.586
Average	0.996	0.598 - 1.659	0.988
Wealthier	0.986	0.546 - 1.778	0.961
Wealthiest	0.752	0.393 - 1.441	0.391
*Location*				
*Urban				
Rural	1.05	0.672 - 1.636	0.835
*Geopolitical zone*				
Northcentral Northeast	1.043	0.612 - 1.775	0.878
Northwest	0.586	0.356 - 0.976	0.036
Southeast	0.972	0.519 - 1.822	0.929
Southsouth	0.459	0.252 - 0.837	0.011
Southwest	0.687	0.359 - 1.318	0.260

*Reference category

**Table 5.** Factors associated with IPTp use.

	OR	95% CI	P-value
*Education*				
*No formal education				
Formal education	1.97	1.706 - 2.275	<0.001
*Wealth quintile*				
*Poorest				
Poorer	1.265	1.065 - 1.504	0.008
Average	1.891	1.564 - 2.286	<0.001
Wealthier	2.353	1.904 - 2.907	<0.001
Wealthiest	3.922	3.109 - 4.947	<0.001
*Location*				
*Urban				
Rural	0.979	0.838 - 1.144	0.791
*Geopolitical zone*				
*Northcentral				
Northeast	1.47	1.206 - 1.792	<0.001
Northwest	1.15	0.960 - 1.382	0.128
Southeast	2.01	1.597 - 2.528	<0.001
Southsouth	1.48	1.219 - 1.807	<0.001
Southwest	1.20	0.987 - 1.469	0.067

*Reference category

## 4 Discussion

The use of ITN and IPTp are two of the most effective measures in reducing the risk of malaria among pregnant women. This study showed a high level of ITN use but a low level of IPTp uptake. This high rate of ITN use may not be surprising, given that bednets have been provided for free, although women may also purchase them or get them from other sources. However, this high ITN utilisation contradicts the findings of a previous study in which low ITN use was reported among pregnant women attending private and public clinics in Lagos State [10]. In addition, previous studies in Nigeria have documented low ITN use among pregnant women in various states, such as North-western Nigeria [18], Imo [19], Northern states [20], Osun [21], and in Edo [8]. However, ownership rates have been high, which does not necessarily translate into use [8,10,18-20]. A Nigerian study conducted in 21 states also reported a low ITN use, of 7.5% [22]. In a review of ITN use during pregnancy by Singh *et al.* [12], the poorest -performing country was Swaziland with an ITN use rate of 1%, followed by Nigeria with an ITN use rate of 5%. Our analysis using the NARHS Plus dataset has shown an ITN use rate of 61.0%. Even though this is still far from the WHO/RBM targets, we can state that this is a great improvement compared to the results of earlier surveys. Our results also showed that ITN use was higher in the northern geopolitical zones compared with the southern zones, a finding consistent with the report of Ankomah [22]. As the majority of communities in the northern zones are mostly rural, this might be an indication of successful community campaigns in rural areas. The high utilisation rates observed might also be attributed to the efforts of local (SuNMaP) as well as international NGOs (JHPIEGO) operating in the country. The activities of these NGOs have contributed to a significant decrease in malaria in Nigeria [23,24]. Support to National Malaria Programme in Nigeria (SuNMaP) in particular has provided technical assistance to the Nigerian National Malaria Elimination Programme (NMEP) to scale up malaria control across ten states (Anambra, Kano, Lagos, Niger, Kat-sina, Ogun, Jigawa, Enugu, Kaduna, and Yobe) from 2008 until 2015. They distributed 12 million long-lasting insecticide-treated nets (LLINs), which increased the coverage in these states from 7% in 2005 to 58% in 2014 [23]. Another international NGO, JHPIEGO, also has community participatory intervention programmes on malaria in pregnancy, and they have worked in Akwa Ibom, Kano, Zam-fara, and Katsina states. Through these programmes, JHPIEGO has distributed ITNs and provided IPTp, which improved ANC attendance and IPTp coverage [24].

Although current IPTp guideline suggests that all pregnant women should receive at least two doses of IPTp, it is evident that all the states in Nigeria are struggling to meet the RBM targets and the 2005 Abuja target in terms of IPTp use. However, for ITN use, Ebonyi, Kwara and Yobe met the 80% target with coverage rates of 87.5, 80.0, and 88.2%, respectively. This is not surprising, given the activities of SuNMaP in some of these states [23]. Low uptake of IPTp may also not be an unusual finding in Nigeria, as several studies have shown that utilisation of IPTp in Africa is poor [25]. Kenya and Malawi are the earliest countries to have started IPTp in Africa, and their coverage has been estimated to be 80.7% and 35.5%, respectively. In addition, Somalia, Benin, Burkina Faso and Congo have IPTp coverage rates of less than 10%, whereas Central Africa and Madagascar have IPTp coverage rates of 11.8% and 12%, respectively. All these countries recorded low coverage for IPTp, despite their high antenatal clinic attendance [26].

What factors might be responsible for this low utilisa-tion and coverage of IPTp? After controlling for other variables in this study, IPTp use depended on education, so-economic status (measured by wealth quintiles) and geopolitical zones. Educated women, wealthier women, women in the north-east, south-east and south-south were more likely to use IPTp. However, it is important to note that education, wealth and location were not associated with ITN use. The only predictor of ITN use identified was geopolitical zone; women in the north-west and south-south were less likely to use ITNs. Although earlier studies have shown that education positively influences health-seeking behaviour (as demonstrated by an Ethiopian study which reported that higher level of education of pregnant women was a significant predictor of ITN use during pregnancy [27]), our findings did not reflect this. However, our findings were similar to those of Ankomah *et al.* [22], who also showed that education was not associated with ITN use. These results suggest that some not-yet-measured socio-cultural factors might be mediating the relationship between education and ITN use in Nigeria. The majority of the women who gave birth in the last 5 years and those who were currently pregnant had formal education. This is not surprising, as literacy rates in Nigeria have increased [15]. Formal education in this context includes Quranic, primary, secondary and higher education. Nigeria has adopted the Universal Basic Education (UBE) scheme, which ensures or stipulates that every child should at least receive primary education. The mean age of the women in this survey was 29 years, which implies that the majority should have undergone the UBE programme. Socioeconomic status as measured by the wealth index was low for the two categories of women [i.e. women who gave birth in the last five years and women who were currently pregnant]. This is in line with previous studies showing that women in Nigeria are not economically empowered and as such do not have access to the basic amenities of life. A Tanzania study also showed that out-of-pocket expenditure for women might be one of the factors causing low ANC utilisation, which in turn will reduce IPTp uptake [28].

Previous studies in Nigeria have identified a lack of health worker training and incorrect knowledge as factors influencing low coverage of IPTp [29]. However, we did not measure or report this in our analysis. Living in urban areas was also not associated with ITN use. Our findings revealed that ITN use was higher in the northern regions; however, IPTp use was also higher in the southern regions. This might be a result of massive community level campaigns for ITN use in the north compared to the south. Earlier studies have shown that pregnant women in the predominantly rural northern zones use ITNs more frequently than pregnant women in the urbanised southern regions [22]. Low IPTp coverage was also reported in Tanzania, and policy and facility factors have been identified as barriers to this low coverage [30]. Earlier studies in Nigeria have documented low IPTp coverage. In Enugu, Onoka *et al.* [29] documented low IPTp use among pregnant women attending antenatal clinics. They further showed that high clinic attendance does not translate into uptake of IPTp.

IPTp scale up will require great efforts, such as effective leadership and governance, which will create more awareness and education, and ensure efficient distribution and utilisation for the benefit of pregnant women. Good health system information will also be required to ensure monitoring and evaluation of IPTp distribution data. Poverty is another bottleneck that affects good health-seeking behaviour. Women who are poor will neither attend ANC services nor receive IPTp. Therefore, female empowerment is also important.

In many healthcare facilities in sub-Saharan Africa, including Nigeria, health workers are regularly overworked and often underpaid and also experience incessant strikes, which might lead to low job satisfaction. Overworked and underpaid staff might show a negative attitude to a pregnant client. In Nigeria, pilot studies on coverage indicators for monitoring and evaluation of IPTp have been conducted. However, good monitoring systems are not in place to ensure efficient utilisation; hand-written registers are still used, which is time-consuming and error-prone [31].

We have used secondary survey data in this analysis and thus could not analyse some variables that have been identified in previous studies, especially health worker characteristics/information, and timing of ANC visits. The responses were self-reported and were not validated from alternative or other sources. To our knowledge, this is the first time that national estimates (by state) of the annual number of pregnancies and the estimated number of live births covered against malaria was made. Our results showed that in 2012, about 3.5 million pregnancies were covered by ITN use while about 2.5 million were covered by IPTp. Compared with the number of live births of about 5.8 million, we note that the country is still far behind in its efforts towards malaria control in pregnancy.

## 5 Conclusions

ITN coverage was higher than previous estimates, even though it is still below the RBM targets. However, IPTp coverage was low despite ANC attendance. Only 4 of 36 Nigerian states achieved the 2010 target of 80% coverage for ITN use. No state achieved the 100% coverage for IPTp. At the present pace, very few states are likely to meet the 2015 target of 100%. The question is thus: if ITN use is high, then why is IPTp uptake low? This suggests that mass distribution of ITNs at antenatal services is a success; however, IPTp services is still lagging behind. If ITNs are distributed during ANC, why are pregnant women not also receiving IPTp? We thus need to determine the barriers that affect IPTp uptake in the delivery of ANC services. In terms of health programmes, efforts should be geared towards increasing ITN utilisation and behaviour change interventions for malaria control in pregnancy in order to meet the RBM targets. Coverage of IPTp showed a higher inequity compared with that of ITNs, with richer and more educated women more likely to receive IPTp than their poorer and less educated counterparts.

## References

[1] Dhiman S, Yadav K, Goswami D, Das NG (2012). Epidemiology and risk analysis of malaria among pregnant women.. Iran. J. Publ. Health.

[2] Taylor SM, van Eijk AM, Hand CC, Mwandagalirwa K (2011). Quantification of the burden and consequences of pregnancy-associated malaria in the Democratic Republic of the Congo.. J. Infect. Dis..

[3] Ezebialu IU, Eke AC, Ezeagwuna DA, Nwachukwu CE (2012). Prevalence, pattern, and determinants of placental malaria in a population of southeastern Nigerian parturi-ents.. Int. J. Infect. Dis..

[4] Rijken MJ, McGready R, Boel ME, Poespoprodjo R (2012). Malaria in pregnancy in the Asia-Pacific region.. Lancet Infect. Dis..

[5] Takem EN (2013). D'Alessandro U: Malaria in pregnancy.. Mediterr. J. Hematol. Infect. Dis..

[6] World Health Organization Evidence Review Group: (2012). Intermittent Preventive Treatment of Malaria in Pregnancy (IPTp) with Sulfadoxine-Pyrimethamine (SP), WHO Headquarters, Geneva, 9-11 July.

[7] Roll Back Malaria: (2008). Guidelines for core population-based indicators..

[8] Wagbatsoma VA, Aigbe EE (2010). ITN utilization among pregnant women attending ANC in Etsako West LGA, Edo state, Nigeria.. Niger. J. Clin. Pract..

[9] Adogu P, Ijemba C (2013). Insecticide treated nets possession and utilization among pregnant women in Enugu Nigeria: A descriptive cross-sectional study.. J. Nat. Sci. Res..

[10] Aina BA, Ayeni FA (2011). Knowledge and use of insecticide treated nets as a malaria preventive tool among pregnant women in a local government area of Lagos state, Nigeria.. J. Appl. Pharmac. Sci..

[11] Federal Ministry of Health Nigeria (FMoH): National HIV/ AIDS and Reproductive Health and Serological Survey (NARHS Plus), 2012.. Abuja, Nigeria;.

[12] Singh M, Brown G, Rogerson SJ (2013). Ownership and use of insecticide-treated nets during pregnancy in sub-Saharan Africa: a review.. Malar. J..

[13] Fagbamigbe AF, Bamgboye EA, Yusuf OB, Akinyemi JO (2015). The Nigeria wealth distribution and health seeking behaviour: evidence from the 2012 national HIV/AIDS and reproductive health survey.. Health Econ. Rev..

[14] StataCorp. (2011). Stata: Release 12.. Statistical Software..

[15] National Population Commission (Nigeria) and ICF International: NPC (2006). Nigeria Household Population Census..

[16] National Population Commission (Nigeria) and ICF International.: (2013). Nigeria Demographic and Health Survey.

[17] Dellicour S, Tatem AJ, Guerra CA, Snow RW (2010). Quantifying the number of pregnancies at risk of Malaria in 2007: A Demographic study.. PLoS Med..

[18] Isah AY, Nwobodo EI (2009). Awareness and utilization of insecticide treated mosquito nets among mothers at a tertiary health institution in north-western Nigeria.. Niger. J. Med..

[19] Iwu RU, Ijioma BC, Egeruoh AS, Awurum IN (2010). Awareness and use of insecticide treated nets among pregnant women attending ante-natal clinic at Federal Medical Center and General Hospital, Owerri, Imo state.. Rep. Opin..

[20] Musa OI, Salaudeen GA, Jimoh RO (2009). Awareness and use of insecticide treated nets among women attending ante-natal clinics in a northern state of Nigeria.. J. Pak. Med. Assoc..

[21] Adeyemi AS, Adekunle DA, Akintola SE (2007). Use and prevalence of insecticide treated mosquito bednets among pregnant population in Oshogbo, Nigeria.. Niger. Med. Pract..

[22] Ankomah A, Adebayo SB, Arogundade ED, Anyanti J (2012). Determinants of insecticide-treated net ownership and utilization among pregnant women in Nigeria.. BMC Publ. Health.

[23] Support to National Malaria Programme in Nigeria (SuNMaP). (2016). Malaria Control in Nigeria.. http://www.malariaconsortium.org/SuNMaP.

[24] JHPIEGO Home Page: http://www.jhpiego.org/en/content/who-we-are.

[25] Brieger WR, Onyido OE, Sexton JD, Ezike VI (1996). Monitoring community response to malaria control using insecticide impregnated bed nets, curtains and residual spray at Nsukka, Nigeria.. Health Educ. Res..

[26] Van Eijk AM, Hill J, Larsen DA, Webster J (2013). Coverage of intermittent preventive treatment and insecticide-treated nets for the control of malaria during pregnancy in sub-Saharan Africa: a synthesis and meta-analysis of national survey data, 2009–11.. Lancet Infect. Dis..

[27] Belay M, Deressa W (2008). Use of insecticide treated nets by pregnant women and associated factors in a pre-dominantly rural population in northern Ethiopia.. Trop. Med. Int. Health.

[28] Mubayazi GM, Magnussen P, Goodman C, Bygbjerg IC (2008). Implementing intermittent preventive treatment for malaria in pregnancy: Review of prospects, achievements, challenges and agenda for research.. Trop. Med. J..

[29] Onoka CA, Hanson K, Onwujekwe OE (2012). Low, coverage of intermittent preventive treatment for malaria in pregnancy in Nigeria: demand-side influences.. Malar. J..

[30] Marchant T, Nathan R, Jones C, Mponda H (2008). Individual, facility and policy level influences on national coverage estimates for intermittent preventive treatment of malaria in pregnancy in Tanzania.. Malar. J..

[31] Crawley J, Hill J, Yartey J, Robalo M (2007). From, evidence to action? Challenges to policy change and programme delivery for malaria in pregnancy.. Lancet Infect. Dis..

